# Differences between xenotransplantation and allogeneic kidney transplantation: the current situation and future challenges in Japan

**DOI:** 10.1007/s10047-025-01506-x

**Published:** 2025-05-02

**Authors:** Soichi Matsumura, Yoichi Kakuta, Yoko Maegawa-Higa, Shota Fukae, Ryo Tanaka, Shigeaki Nakazawa, Kazuaki Yamanaka, Shuji Miyagawa, Norio Nonomura

**Affiliations:** 1 Department of Urology, Osaka University Graduate School of Medicine, 2-2 Yamada-oka, Suita, Osaka 565-0871 Japan; 2 Department of Pediatric Surgery, Osaka University Graduate School of Medicine, Suita, Osaka Japan

**Keywords:** Kidney transplantation, Xenotransplantation, Allotransplantation

## Abstract

Kidney transplantation is the only curative option for patients with chronic renal failure, significantly improving their survival and quality of life. However, this treatment remains limited by the shortage of organ donors. The shortage of kidney donors remains a serious problem all over the world, and is particularly severe in Japan. While advancements in immunosuppressive therapies and histocompatibility testing have improved outcomes in allogeneic kidney transplantation, the rising number of dialysis patients has worsened the gap between the demand for and supply of suitable donor organs. In response to this pressing need, xenotransplantation has gained attention as a promising alternative solution. Recent progress driven by gene-editing technologies, including CRISPR-Cas9, has facilitated the development of genetically modified pigs suitable for potential human transplantation. This review provides an overview of the key differences in immune response and infection risks between xenogeneic and allogeneic kidney transplants. In addition, it comprehensively examines the challenges and potential of xenogeneic kidney transplantation from multiple perspectives, including differences in immunosuppressive therapies between allogeneic and xenogeneic transplantation. We also discuss the feasibility of xenogeneic kidney transplantation as a solution to the organ shortage in Japan and present directions for addressing challenges toward clinical application. We hope this review will provide valuable insights into the potential of xenogeneic kidney transplantation as a new treatment option for chronic renal failure and contribute to efforts to address the donor shortage problem in Japan.

## Current status of allogeneic kidney transplantation in Japan

**Fig. 1:**
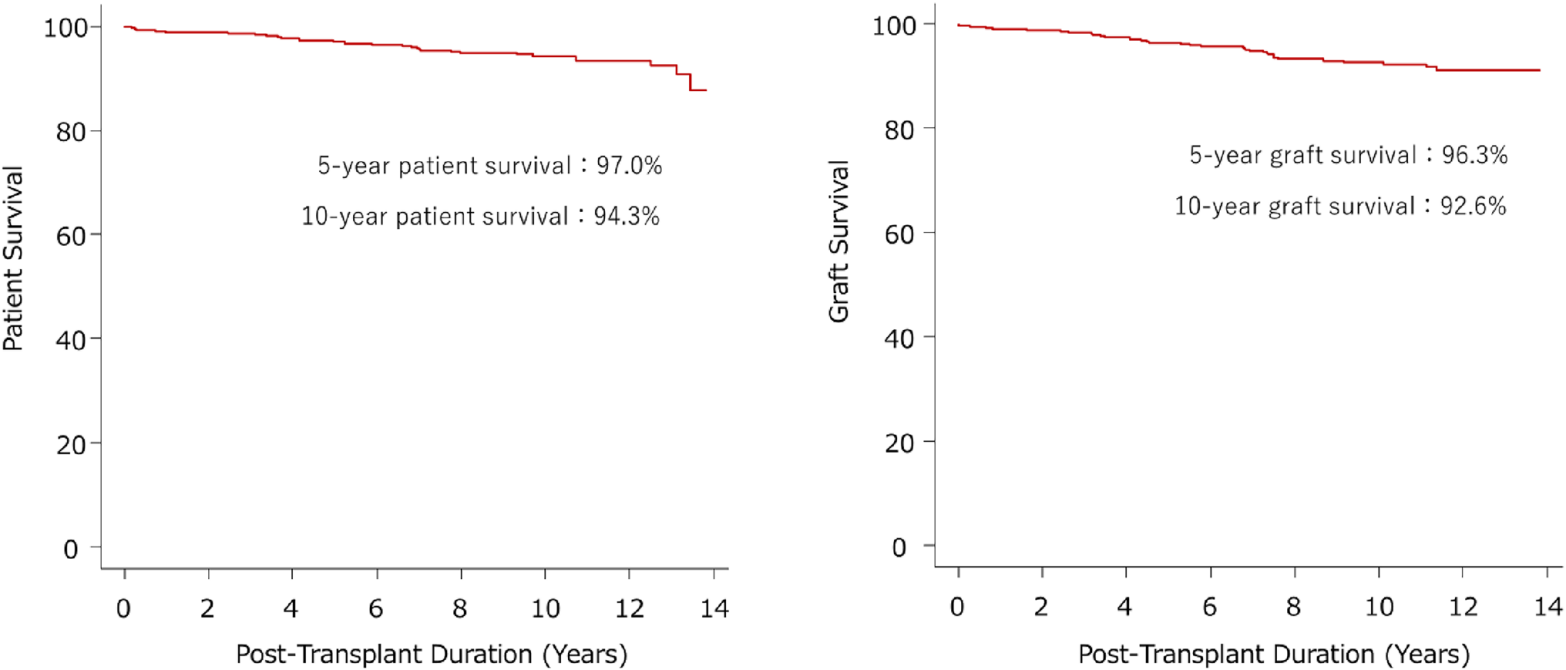
Patient and graft survival rates for living-related kidney transplantation performed by the University of Osaka Kidney Transplant Group

Patients with chronic renal failure can undergo renal replacement therapy such as hemodialysis, peritoneal dialysis, and kidney transplantation. The characteristics of these treatments are summarized in Table 1. Among these, kidney transplantation is the only curative treatment for chronic renal failure. Recent advances in immunosuppressive therapies and histocompatibility testing have significantly improved transplantation outcomes, transforming it into a more reliable and effective treatment. According to the result of the ERA Registry Annual Report, the 5-year survival rate for patients on dialysis was reported to be 42.3%, whereas it was 86.6% for recipients of deceased donor kidney transplants and 94.4% for recipients of living donor kidney transplants [1]. Similar outcomes have been reported in Japan [2], and the outcome of our transplant group is shown in Fig. 1. In addition, the quality of life of transplant recipients has profoundly improved [3]. Hemodialysis requires > 50 h of treatment per month, imposing substantial time and lifestyle constraints on patients. In contrast, transplant recipients are free from these time burdens, significantly increasing their chances of returning to work or school. Moreover, they also experience fewer dietary restrictions and avoid the taste disturbances commonly associated with hemodialysis. For young women, kidney transplantation restores the possibility of conceiving, benefit that is reduced with dialysis. In children, transplantation removes critical barriers to both physical and emotional development, allowing them to thrive in a way that dialysis cannot support. From an economic perspective, kidney transplantation has a significant impact and contributes to reducing healthcare costs compared to hemodialysis. This cost-effectiveness is evident even in high-risk transplant cases, such as ABO-incompatible kidney transplantation, where the long-term benefits and cost savings continue to outweigh the initial risks and expenses [4].

**Table 1:** Comparison of kidney transplantation, hemodialysis, and peritoneal dialysis: impact on patient lifestyle and treatment outcomes in Japan

	Kidney transplant	Hemodialysis	Peritoneal dialysis
Kidney function	Near normal level (60–70%)	Renal failure	
Surgery required	Kidney transplant surgery (general anesthesia)	Shunt operation (local anesthesia)	Peritoneal catheter insertion surgery
Number of hospital visits	Once/1–2 months	3 times/week	1 time/month
Subjective symptoms due to treatment	Patients generally report minimal to no symptoms related to the transplant	Many patients experience moderate-to-severe symptoms during and after treatment (e.g., fatigue, cramps)	Patients may experience mild-to-moderate symptoms such as discomfort from the catheter or abdominal distension
Immunosuppressant (drug)	Required	Not required	Not required
Dietary and fluid restrictions	Moderate dietary restrictions	Numerous (protein, water, salt, potassium, phosphorus, etc.)	A little more than usual (protein, water, salt, phosphorus, etc.)
Travel, business trip	Easier	Difficult (securing outpatient dialysis facilities)	Moderate (preparation and transport of dialysis fluid and equipment)
Delivery	Possible	Difficult	Difficult
Sport	Possible with precautions	Limited	Care must be taken to avoid abdominal pressure
Take a bath	Possible	Showering is preferred after dialysis	Catheter needs to be protected
Reintegration rate	High	Moderate probability	Relatively high
Other benefits	Freedom from restrictions caused by dialysis	The most established treatment method in Japan, where medical care is always provided	More flexible than hemodialysis

According to a survey conducted by the Statistical Survey Committee of the Japanese Society for Dialysis Therapy, as of the end of 2021, there were 349,700 patients undergoing dialysis in Japan, representing 2786 patients per million people, and this number continues to increase [5, 6]. Despite this growing demand for renal replacement therapy, only 1773 kidney transplants were performed in Japan in 2021, of which 1648 and 125 were from living and deceased donors, respectively. In contrast, approximately 25,000 kidney transplantations are performed annually in the United States [7]. The fact that transplant recipients in Japan represent an only 0.5% of the total dialysis population highlights a critical disparity, reflecting a severe shortage of organ donors.

To address this critical situation, studies are being conducted in the fields of regenerative medicine and bioengineered organogenesis, where xenotransplantation is being explored as a potential solution. If successfully developed and applied in clinical practice, xenotransplantation could mitigate the chronic donor shortage and represent a transformative milestone in the treatment of chronic renal failure worldwide, especially in Japan.

## Current status of xenogeneic kidney transplantation

Xenogeneic kidney transplantation has seen significant progress in the past decade, largely due to advancements in gene-editing technologies like CRISPR (Clustered Regularly Interspaced Short Palindromic Repeats) and Cas9 (CRISPR-associated protein 9) [8]. Revivicor successfully developed 10-gene-edited (10-GE) pigs that underwent 10 genetic modifications [9]. This section focuses on xenogeneic kidney transplantations conducted after 2021.

In September 2021, Montgomery et al. performed a groundbreaking xenotransplantation, transplanting porcine kidneys with a knockout of the α-1,3-galactosyltransferase (αGal) gene into brain-dead patients, a significant step toward addressing the organ shortage crisis [10]. In addition, to mitigate immune rejection, they also transplanted the porcine thymus gland to generate immune cells that could help the recipients tolerate the xenogeneic organs. The transplanted kidneys were planned to be removed after 54 h, during which no signs of rejection were observed, and creatinine levels showed improvement, indicating functional compatibility between the porcine kidneys and the human recipients. However, despite these promising results, this study has some limitations that must be addressed. Xenoantigens involved not only αGal but also other antigens, such as Neu5Gc, produced by cytidine monophospho-N-acetylneuraminic acid hydroxylase (CMAH), and Sda, produced by β1,4-N-acetylgalactosaminyl transferase 2 (β4GalNT2). Complete immune compatibility may require genetic modification of all three enzymes. However, in this study, the pigs used had only the αGal gene deleted, which leaves room for further investigation into the full spectrum of immune response. Another limitation lies in the fact that the recipient’s native kidneys were not removed, raising uncertainty about whether the observed urine production originated from the porcine kidneys or the recipient’s own kidneys. This ambiguity limits the ability to conclusively assess the functional viability of the xenotransplanted kidneys. Nonetheless, this study represents a critical milestone in the development of xenotransplantation, bringing the field closer to realizing its clinical potential.

In January 2022, Porrett et al. made a significant advancement in xenotransplantation by directly addressing a critical limitation of earlier studies. In their approach, both native kidneys were surgically removed from brain-dead patients prior to the transplantation of porcine kidneys eliminating any ambiguity regarding the source of urine production observed post-transplantation [11]. The porcine kidneys used in this study were sourced from Revivicor’s 10-GE pigs, which had additional genetic modifications. In addition to the knockout of the αGal, CMAH, and β4GalNT2 genes, these pigs were engineered with the introduction of complement regulatory proteins and growth inhibitors, designed to further minimize the immune response and promote long-term graft survival. In this trial, two porcine kidneys were transplanted and closely monitored over a 74-h period. Remarkably, there were no signs of rejection, and the porcine kidneys successfully produced urine, providing compelling evidence of functional compatibility. Despite these promising early results, the study revealed a critical challenge: by the end of the study period, there was no significant improvement in kidney function. This may have been because the brain-dead patients had been hemodynamically unstable for 5 days prior to the procedure, a condition likely to have impaired the ability of the transplanted organs to fully restore renal function.

In March 2024, the world’s first genetically modified pig kidney was successfully transplanted into a patient with renal failure. Although the patient passed away 7 weeks later, this groundbreaking trial represents a major step forward in the field of xenogeneic kidney transplantation [12].

This outcome underscores the need for further research to determine whether the 10-GE pig kidneys can effectively suppress rejection and achieve durable renal function when transplanted into living human patients, where the physiological environment would be more stable. Nevertheless, despite the remaining challenges, recent developments in xenotransplantation using genetically modified pigs suggest that this innovative approach is rapidly becoming a viable option for clinical application. As the field continues to progress, it holds the potential to revolutionize the treatment landscape for end-stage renal disease and address the growing organ shortage crisis on a global scale.

## Differences in immune responses in allotransplantation and xenotransplantation

The main difference between allogeneic and xenogeneic immune responses lies in the reaction to antigens. In allotransplantation, immune responses are primarily triggered by antibodies against ABO blood group antigen or donor-specific HLA antigens. These issues can be managed through desensitization therapies, including intravenous immunoglobulin, plasma exchange, rituximab, and so on. However, the immune response in xenotransplantation is markedly more intense, primarily due to the presence of xenogeneic carbohydrate antigens. Despite significant advancements in genetic engineering, including modifications aimed at reducing the expression of these antigens, the immune system’s reaction to xenotransplanted tissues remains substantially stronger than in allotransplantation [13].

In xenogeneic transplantation, differences in the complement and coagulation systems have historically posed significant challenges. These issues have been partially addressed by knocking in human genes into pigs. Recently, “Innate immunity” is one of the most formidable obstacles in xenotransplantation, particularly its cellular components [14]. Human natural killer (NK) cells are highly reactive to xenografts due to insufficient inhibitory signaling between human NK cells and the donor’s major histocompatibility complex (MHC) molecules [15]. This insufficient signaling results in the activation of NK cells, leading to xenograft destruction. Current strategies focus on blocking NK cell activation and enhancing inhibitory receptor signals to suppress NK cell xenoreactivity. For instance, inhibiting receptors such as CD2 and NKG2D, along with expressing human leukocyte antigen (HLA) class Ib molecules on xenografts, has shown promise in significantly reducing NK cell-mediated xenoreactivity [16, 17]. However, innate immune responses are not limited to NK cells. Human macrophages can directly phagocytose porcine cells without antibodies or complement, contributing to solid-organ xenograft rejection. The incompatibility between porcine CD47 and human signal regulatory protein α (SIRPα) plays a key role in macrophage-mediated rejection [18]. By introducing human CD47 into porcine cells, researchers have successfully protected these cells from macrophage attack, a modification already incorporated into and Revivicor’s 10-GE [19].

Another critical player in xenotransplant rejection is neutrophils. These immune cells, which interact closely with the endothelial cells lining blood vessels, can directly recognize and react with xenogeneic endothelial cells. For example, porcine aortic endothelial cells have been shown to activate human neutrophils, initiating a robust inflammatory response. Research efforts aimed at controlling neutrophil activity in xenotransplantation are still in their infancy, presenting a significant hurdle for the successful clinical application of xenotransplantation [14, 20].

In terms of acquired immunity, xenotransplantation triggers a strong response from CD4-positive T cells, which primarily recognize porcine swine leukocyte antigen (SLA) class I and II molecules. These T cells mediate their attack on porcine cells through the Fas–Fas ligand (FasL) pathway, inducing apoptosis in the xenogeneic tissues. Research into modulating this pathway has led to experimental strategies, such as the co-transplantation of a porcine thymus alongside the kidney to induce tolerance by promoting immune cell education within the thymus [21].

A crucial focus of immune suppression in xenotransplantation is the inhibition of CD40/CD154 co-stimulation, which is central to T-cell activation. Although anti-CD154 antibodies have demonstrated efficacy in reducing immune reactions, their clinical application remains constrained by the risk of thromboembolic complications due to platelet activation [22]. Ensuring the safe use of these agents will be vital for advancing xenotransplantation toward routine clinical practice.

Overall, while considerable progress has been made in mitigating both innate and acquired immune responses, xenotransplantation remains an area of ongoing research. The refinement of genetic modifications and immunosuppressive strategies will be essential for overcoming the remaining immunological barriers and achieving long-term xenograft survival in humans.

## Differences in immunosuppressive therapy in allogeneic and xenogeneic kidney transplantation

**Fig. 2:**
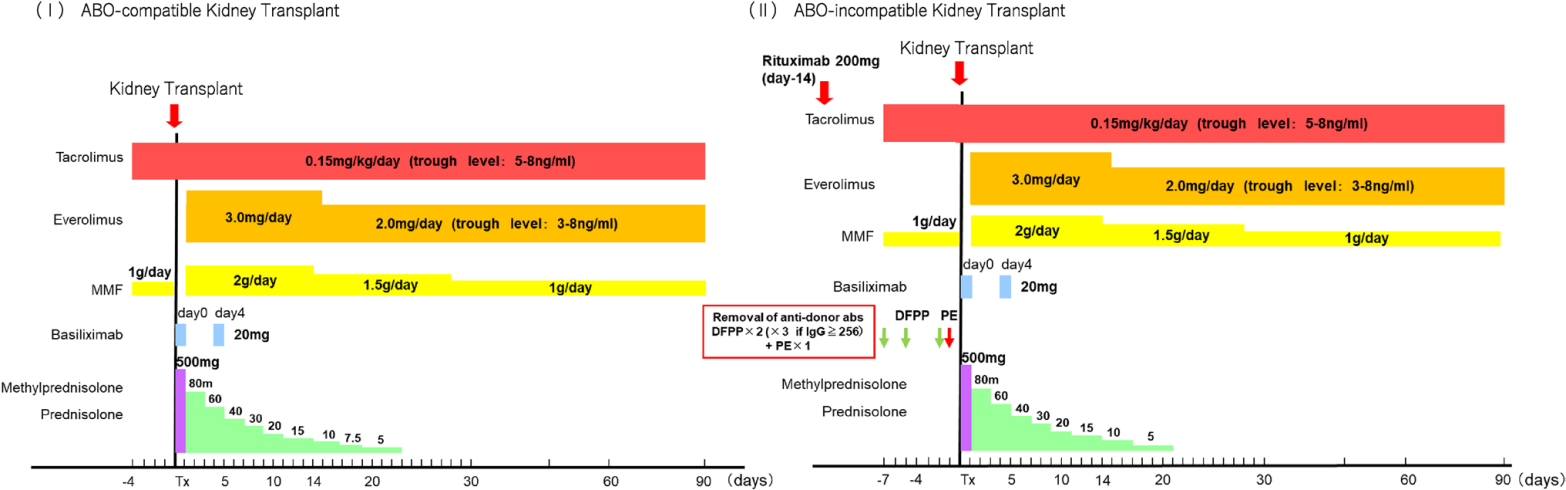
Immunosuppressive protocol in University of Osaka

In allogeneic kidney transplantation, immunosuppressive therapy typically involves a multi-drug regimen aimed at reducing side effects. The immunosuppressive protocol used in our hospital is shown in Fig. 2. It includes the extended-release formulation of tacrolimus (a calcineurin inhibitor), mycophenolate mofetil (an anti-metabolic agent), steroids, basiliximab (an anti-CD25 monoclonal antibody), and everolimus (an mTOR inhibitor), with early steroid withdrawal. In ABO-incompatible or donor-specific anti-HLA antibody positive kidney transplantation, desensitization therapy is used to eliminate antibodies and suppress B-cell activity. Our desensitization protocol includes high-dose IVI intravenous immunoglobulin (IVIG), rituximab (an anti-CD20 monoclonal antibody), and antibody removal therapy. Generally, desensitization therapy is based on either high-dose IVIG or antibody removal therapy combined with low-dose IVIG, often supplemented by rituximab. The effectiveness of new therapeutic agents has also been increasingly reported [23]. In cases of T-cell-mediated rejection, rabbit anti-human thymocyte immunoglobulins can be used, and plasma exchange and IVIG can be used in cases of antibody mediated rejection.

However, immunosuppressive therapy for xenotransplantation remains poorly defined owing to the limited number of human clinical trials. One of the few documented cases occurred in January 2022; it involved xenotransplantation in a patient with severe heart failure [24]. In this case, rituximab and anti-thymocyte globulin were used to deplete the B and T cells, and a C1 esterase inhibitor (human C1-inactivator) was administered to block complement activation. As discussed earlier, the inhibition of the CD40/CD154 co-stimulatory pathway is crucial for managing immune responses in xenotransplantation [25]. In this study, a humanized anti-CD40 monoclonal antibody (KPL-404) was used to block this pathway. The maintenance immunosuppressive regimen comprised mycophenolate mofetil, KPL-404, and steroids. In recent years, a randomized, double-blind, placebo-controlled trial in humans has been conducted, suggesting the efficacy of chronic administration of KPL-404 [26].

One notable distinction between allogeneic and xenogeneic transplants is the unique role of B-1b cells, a specialized subset of B cells resistant to calcineurin inhibitors (CNI) [27]. These cells play a pivotal role in the immune response by producing natural antibodies specifically targeting xenogeneic carbohydrate antigens. The persistence of these cells, despite immunosuppressive therapy, poses a substantial challenge to successful xenotransplantation. In addition, the application of IVIG for treating rejection in xenotransplantation is also controversial [28]. IVIG’s mechanisms of action may behave differently in the context of xenografts, potentially interacting with donor-specific cells in ways that are not fully understood.

Currently, no standardized immunosuppressive regimen has been developed specifically for xenotransplantation, resulting in a significant deficiency in the ability to prevent rejection and ensure xenograft survival. The intricacies of xenogeneic immune responses, combined with the unique antigenic profile of xenografts, necessitate further research to identify therapeutic strategies capable of promoting durable, long-term xenograft survival. Establishing robust immunosuppressive protocols that address these challenges is critical for the successful clinical application of xenotransplantation, and achieving this goal will require a deeper understanding of both innate and adaptive immune mechanisms involved in xenograft rejection.

## Differences in infections between allotransplantation and xenotransplantation

Primary infectious concerns in allogeneic kidney transplantation include cytomegalovirus (CMV), Epstein–Barr virus (EBV), and BK virus. Cytomegalovirus, a herpesvirus with high seroprevalence, can lead to serious complications, such as enteritis and retinitis, in immunocompromised kidney transplant recipients, often necessitating preemptive treatment or prophylaxis [29]. Epstein–Barr virus, commonly transmitted via human saliva, is present in more than 95% of adults who develop antibodies [30]. In the context of transplantation, EBV can lead to post-transplant lymphoproliferative disorders, a potentially life-threatening condition characterized by uncontrolled B-cell proliferation, particularly in patients on intensive immunosuppressive regimens [31]. Similarly, the reactivation of latent BK virus due to immunosuppression remains a critical concern, as it can lead to BK nephropathy, severely compromising renal graft function [32].

In xenotransplantation, a significant infectious risk centers around porcine endogenous retroviruses (PERVs), with the potential for cross-species transmission being an initial barrier to clinical application [33]. However, advancements in gene-editing technology, particularly by biotechnology firms such as eGenesis, have successfully eliminated PERVs from porcine genomes, effectively mitigating this risk [34]. Studies involving patients who have received porcine tissue or cell transplants have shown no evidence of PERV infection, suggesting that the risk of PERV transmission to humans is likely low, though ongoing monitoring remains crucial [35].

Another major infection in xenotransplantation is porcine cytomegalovirus (PCMV), which has been associated with significantly reduced survival rates in xenotransplanted organs. Research involving baboon models has shown that PCMV-infected porcine organs exhibit markedly shorter survival times, highlighting the need for stringent screening and pathogen elimination processes [36]. Such findings underscore the potential of PCMV to compromise xenograft longevity, particularly in heart xenotransplants, where PCMV has been documented in human recipients, underscoring the virus’s potential impact on graft viability [24, 37, 38].

Infectious diseases in xenotransplantation were a key topic at the 3rd WHO World Congress on Regulatory Requirements for Xenotransplantation Clinical Trials in 2018. The experts highlighted the necessity for a global infrastructure dedicated to monitoring and responding to potential infectious disease outbreaks related to xenotransplantation. The establishment of international laboratory networks for coordinated pathogen detection and surveillance was also prioritized. Meeting these global regulatory and collaborative requirements is essential to advancing xenotransplantation into clinical practice while safeguarding public health [39].

## Conclusion

Allogeneic transplantation has been established as a viable, long-term solution, with significant improvements in immunosuppressive protocols and histocompatibility testing leading to enhanced patient survival rates and quality of life compared to dialysis. However, in Japan, a severe shortage of organ donors persists, necessitating the exploration of alternative solutions. Xenogeneic kidney transplantation, made possible by gene-editing technologies, has shown promise, presenting the potential for genetically modified pig organs suitable for human transplantation. Further basic research on immune-related mechanisms is essential to develop better genetically modified pigs, which will be the key to successful xenotransplantation in the future. At the same time, xenotransplantation faces unique immunological and infectious challenges, including immune responses to xenogeneic antigens, issues from innate immune cells, and risks associated with porcine-specific pathogens like PCMV.

For clinical application, robust immunosuppressive strategies, global infection monitoring, and international laboratory collaboration are essential. Addressing these complex challenges may enable xenotransplantation to alleviate organ shortages, offering a transformative solution for renal replacement therapy. Although Japan still faces many challenges compared to other countries, it is anticipated that with the development of appropriate systems and infrastructure in the future, xenotransplantation could become a feasible and widely adopted treatment option.

This review is based on a paper published in the Japanese Journal of Artificial Organs, written in Japanese.

## Data Availability

No new data were generated or analyzed in this study.
